# Closing the Digital Divide in Speech, Language, and Cognitive Therapy: Cohort Study of the Factors Associated With Technology Usage for Rehabilitation

**DOI:** 10.2196/16286

**Published:** 2020-02-11

**Authors:** Michael Munsell, Emily De Oliveira, Sadhvi Saxena, Jason Godlove, Swathi Kiran

**Affiliations:** 1 The Learning Corp Newton, MA United States; 2 Aphasia Research Laboratory, Speech Language and Hearing Sciences Boston University Boston, MA United States

**Keywords:** aphasia, stroke, traumatic brain injury, technology

## Abstract

**Background:**

For stroke, traumatic brain injury (TBI), and other neurologic conditions associated with speech-language disorders, speech and language therapy is the standard of care for promoting recovery. However, barriers such as clinician time constraints and insurance reimbursement can inhibit a patient’s ability to receive the support needed to optimize functional gain. Although digital rehabilitation has the potential to increase access to therapy by allowing patients to practice at home, the clinical and demographic characteristics that impact a patient’s level of engagement with technology-based therapy are currently unknown.

**Objective:**

This study aimed to evaluate whether the level of engagement with digital therapy differs by various patient characteristics, including age, gender, diagnosis, time from disease onset, and geographic location (urban vs rural).

**Methods:**

Data for patients with stroke or TBI that initiated the use of Constant Therapy, a remotely delivered, cloud-based rehabilitation program for patients with speech-language disorders, were retrospectively analyzed. Only data from therapeutic sessions completed at home were included. The following three activity metrics were evaluated: (1) the number of active weeks of therapy, (2) the average number of active therapy days per week, and (3) the total number of therapeutic sessions completed during the first 20 weeks of program access. An *active* day or week was defined as having at least one completed therapeutic session. Separate multiple linear regression models were performed with each activity measure as the dependent variable and all available patient demographics as model covariates.

**Results:**

Data for 2850 patients with stroke or TBI were analyzed, with the average patient completing 8.6 weeks of therapy at a frequency of 1.5 days per week. Contrary to known barriers to technological adoption, older patients were more active during their first 20 weeks of program access, with those aged 51 to 70 years completing 5.01 more sessions than patients aged 50 years or younger (*P*=.04). Similarly, patients living in a rural area, who face greater barriers to clinic access, were more digitally engaged than their urban counterparts, with rural patients completing 11.54 more (*P*=.001) sessions during their first 20 weeks of access, after controlling for other model covariates.

**Conclusions:**

An evaluation of real-world data demonstrated that patients with stroke and TBI use digital therapy frequently for cognitive and language rehabilitation at home. Usage was higher in areas with limited access to clinical services and was unaffected by typical barriers to technological adoption, such as age. These findings will help guide the direction of future research in digital rehabilitation therapy, including the impact of demographics on recovery outcomes and the design of large, randomized controlled trials.

## Introduction

### Background

An estimated 795,000 people in the United States have a stroke each year, making it the fifth leading cause of death and a major source of disability in adults [1]. Nearly one-third of stroke survivors present with aphasia, an acquired disorder of language processing that can affect speech comprehension, expression, reading, or writing [2,3]. In addition to negatively impacting a patient’s quality of life and ability to participate in their community, poststroke aphasia is also associated with significantly higher rates of mortality, length of hospital stay, and utilization of health care services [4-7]. From 2006 to 2014, the United States experienced a 53% increase in emergency room visits and hospitalizations attributed to another cause of aphasia, traumatic brain injury (TBI) [8]. When evaluated independently, the incidence of TBI deaths decreased by 6% over the same period, indicating that a higher number of people are living post-TBI injury. Similar to stroke, survivors of TBI can experience decreased speech and cognitive function resulting from both the initial impact and the secondary cerebral damage caused by inflammation [9]. For stroke, TBI, and other neurologic conditions resulting in problems with speech and language comprehension (eg, brain tumors and some progressive neurological conditions such as dementia), speech and language therapy (SLT) is the standard of care for promoting functional recovery. A growing body of literature indicates that persons with speech-language disorders continue to improve their language and communication abilities when treatment is continued several months post disease onset [2]. However, after a limited number of therapy sessions immediately following injury, clinician time constraints, insurance reimbursement, and patient fatigue can inhibit a patient’s ability to receive the support they need to maintain gains in functional recovery [10].

One way to offset this lack of sufficient therapy is to enable patients to engage in home practice through technology-based therapeutic programs. Digital therapy delivered via computer, tablet, or smartphone has demonstrated an ability to aid in a patient’s recovery with a similar degree of functional improvement as traditional in-person techniques [11-16]. One such program is called Constant Therapy, a remotely delivered, cloud-based rehabilitation program for patients with speech and cognitive deficits caused by brain injury. Patients who used Constant Therapy at home were able to achieve similar improvements in accuracy on language and cognitive exercises to patients using the app with a clinician. However, patients using the program at home mastered these tasks more quickly (6 days vs 12 days; *P*<.001) because of performing their exercises more frequently [16].

### Objective

Although technology-based rehabilitation programs have the potential to increase access to therapy and promote functional recovery for patients with brain injury, technology may also prove to be a barrier in certain instances. A recent survey of patients using tablet-based poststroke rehabilitation found that device and system issues (eg, unreliable connections, exercise speed, and difficulty using a touchscreen) and the patient’s general comfort level with technology limited their use of the platform [17]. However, these findings were from a small sample of patients in the acute care setting. To understand the feasibility of scaling the delivery of remote therapy for home practice across a large, heterogeneous population, it is important to understand the usage of technology for rehabilitation outside the clinic. In the analysis presented here, we retrospectively examined the usage of the Constant Therapy program across individuals with stroke or TBI and evaluated whether the level of digital engagement differed by various demographic characteristics collected upon account creation. It was hypothesized that known barriers to technological adoption, including older age and a more rural location [18,19], would decrease a patient’s overall usage of the computer-based rehabilitation program, including the number of therapeutic exercises completed, the average frequency of therapeutic sessions, and the total duration of therapy. The information gained from our study could help clinicians understand the expected usage of remotely delivered rehabilitation and enable them to evaluate the feasibility of recommending digital therapy based on high-level patient characteristics.

## Methods

### Study Design and Patients

This study is a retrospective analysis of data collected from patients with stroke or TBI who initiated the use of Constant Therapy during a 40-month period from October 2016 to January 2019. Although it was not required that a patient be formally diagnosed with aphasia or another speech-language disorder, all patients included in this analysis endorsed having a language or cognitive deficit upon account creation. Constant Therapy is a subscription-based platform and is available for download on the iTunes and Google Play stores. Either a clinician set up an account for a patient or the patient created an account after downloading the program themselves. New users were asked to self-select which areas of therapy they felt they needed improvement on, and initial exercises were assigned based on these reported deficits. Before initial account sign in, users were presented with a written description of the user license agreement, where they had to electronically consent to the use of their exercise and therapy performance for scientific and research purposes. Users were also asked to provide information about their demographics, including age (in years), gender, diagnosis, and time since injury. Zip code–level location data were approximated according to the Internet Protocol (IP) address associated with account creation. The mapping of location data to an urban or rural setting was then determined using a crosswalk made publicly available by the US Federal Office of Rural Health Policy, which identifies nonmetropolitan counties and rural census tracts based on zip code.

The intention of this study was to evaluate the usage patterns of digital therapy during home practice; therefore, only users with active home use and only therapeutic sessions completed without the aid of a clinician were eligible for inclusion in the analysis. If a patient was working directly with a clinician throughout the duration of their therapy, a clinician may have reviewed a patient’s progress periodically in between home-based sessions. A patient was only required to have at least one therapeutic session outside the clinic (N=2850) because low utilization rates (eg, 1-2 therapeutic sessions) are of interest to the study hypothesis and help determine the full range of expected digital therapy use across a large sample.

**Figure 1:**
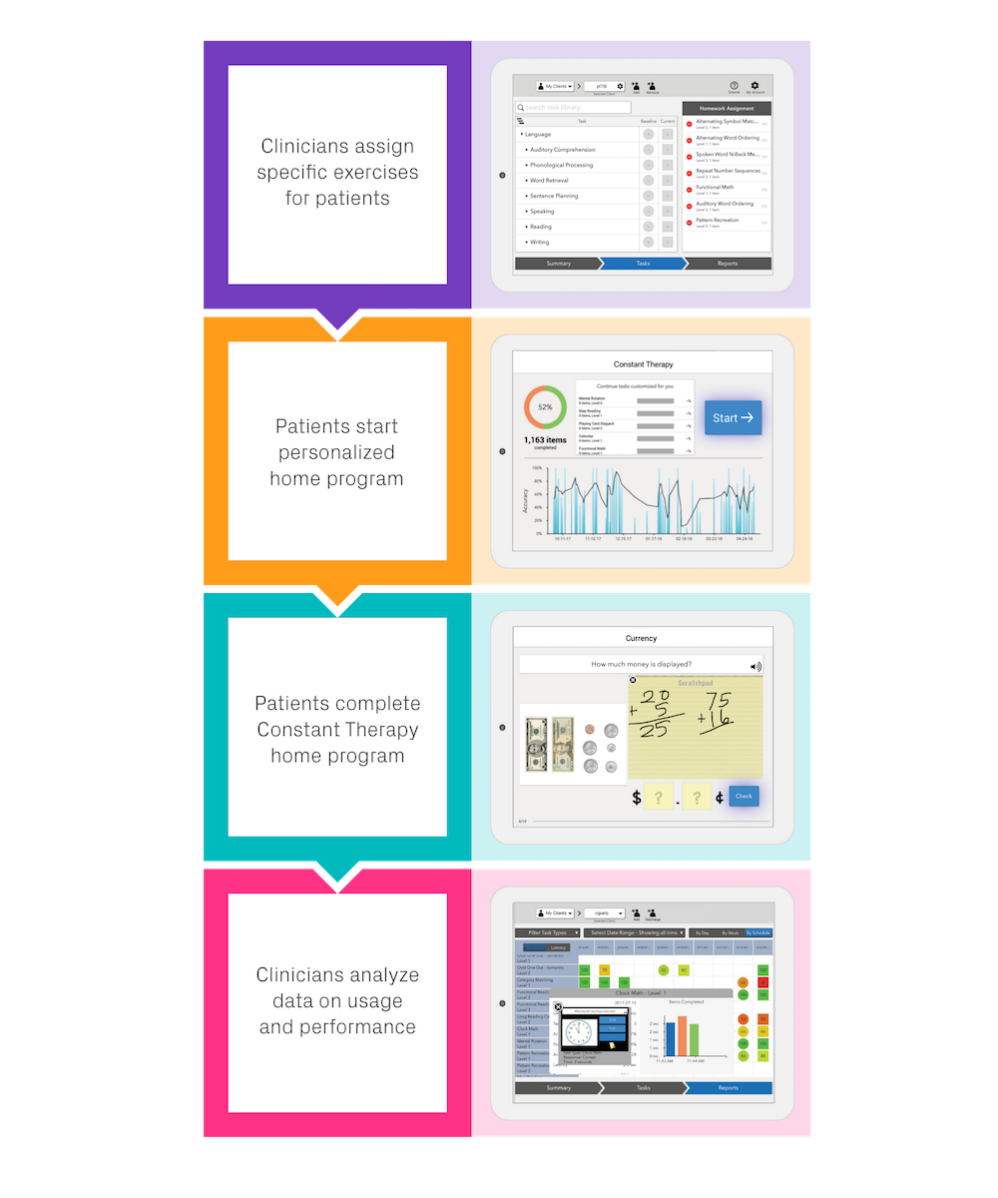
Constant Therapy overview.

During a home-based therapy session, patients practiced exercises in increasing order of difficulty. As a patient worked through the therapeutic schedule, assigned exercises dynamically adapted to each patient’s individual progress. Therefore, although a clinician may be involved in the initial setup of a patient’s therapeutic regimen, the Constant Therapy platform curates a program that continuously identifies and addresses an individual’s recovery needs, enabling patients to practice and advance independently (Figure 1) [15,20].

### Study Ethics Approval

All data from patients’ devices were anonymized upon collection. This project was considered an Institutional Review Board (IRB) exempt retrospective analysis by Pearl IRB (#17-LNCO-101) under Title 45 Code of Federal Regulations 46.101(b) category 2.

### Data Collection

Data were collected using the Constant Therapy platform, which includes more than 80 evidence-based SLT exercises with varying levels of difficulty, for a total of 244 individual exercises. The exercises fall in the domains of *language* (naming, comprehension, speaking, reading, and writing) and *cognitive skills* (attention, executive skills and problem solving, mental flexibility, memory, and visuospatial skills).

As a patient completed therapeutic exercises on their mobile device, the program recorded performance data (task accuracy and latency) and all session activities, including usability logs, time stamps, and item completion indicators. Data were stored in a database and were cleaned before analysis. Missing data may result from various scenarios, including technical issues and patients not completing an assigned therapeutic session. To minimize the impact of missing data on the results of this analysis, we only included therapeutic sessions where the majority (ie, more than one-half) of assigned exercises were completed.

### Statistical Analysis

#### Statistical Methods

Patient demographics were analyzed using descriptive statistics and included patient diagnosis (stroke vs TBI), presence of a chronic condition (>6 months from disease onset), age group at the time of account creation (<50 years, 51-70 years, and >70 years), gender, and a binary indicator set to a value of 1 if a patient lived in a rural census tract as determined by zip code. Analyses of the following three measures of activity were conducted for the full study sample using descriptive statistics: (1) the number of active weeks of therapy, (2) the average number of active days per week, and (3) the total number of therapeutic sessions completed during the first 20 calendar weeks of using Constant Therapy (defined as shown in Textbox 1). To examine the impact of patient demographic characteristics on usage patterns, 3 separate multiple linear regression models were performed with each activity measure as the dependent variable. Model covariates included all available patient demographics listed previously. A multiple linear regression model was determined to be the most appropriate methodological approach for our analysis, given that several factors may determine a patient’s usage of digital therapy, and evaluating the effect of each factor independent of other demographic characteristics is needed for interpretability. All statistical analyses were conducted using the Python programming language and the open-source Statsmodel package [21].

**Activity measure definitions.**

1. Number of active weeks of therapy: The sum of all active weeks for a given patient during the study window. An *active* week was defined as a week with at least one therapy session completed. This measure gauges the total duration of therapy for a given patient while excluding events such as vacations and missed therapy days.
2. Average number of active days per week: An *active* day was defined as a day with at least one therapy session completed. To derive this metric, the total number of active therapy days for a given patient during the study window was divided by their total number of active weeks of therapy. This metric gauges how frequently therapy was performed, on average, during weeks with active therapy.
3. Total number of therapeutic sessions completed during the first 20 calendar weeks of using Constant Therapy: This metric gauges the number of therapy sessions completed for each patient over a fixed period (20 calendar weeks following a patient’s first active session)

#### Power

The number of Constant Therapy users who reside in a rural location was small in the study sample (N=226) relative to the number of nonrural users (N=2624), a finding that is in line with known barriers to technological adoption [19]. To determine if this available sample size was sufficient to estimate a statistically significant difference in digital therapy usage by geographic location, we conducted a *t* test power analysis and varied the effect size level according to Cohen suggested values (ie, small effect=0.2, medium effect=0.5, and large effect=0.8) [22]. The available sample of rural patients was considered sufficient to achieve 80% power to correctly reject the null hypothesis with an alpha of .05 and a small effect size. The validity of sample sizes resulting from age group stratification was also found to be sufficient under the same criteria using a one-way analysis of variance (ANOVA) *F* test power analysis, assuming three age groups. Furthermore, our full sample was sufficient for achieving 80% power with an alpha of .05 in a linear model with 5 degrees of freedom. On the basis of these tests, we concluded that our sample sizes were sufficient for the proposed analysis, specifically, evaluating whether known barriers to technological adoption (age and rural location) impact a patient’s engagement with digital rehabilitation.

#### Sensitivity Analysis

A sensitivity analysis was conducted to understand the robustness of our findings on geographic location. Specifically, propensity score matching was used to create an equally sized sample of patients who lived in an urban setting but did not statistically differ from the full rural sample in terms of age, gender, time from disease onset, diagnosis, or US state of residence. The difference in each of the 3 activity measures between the 2 groups was evaluated using one-way ANOVA. Propensity score matching was completed using the R statistical package using the nearest neighbor method of matching [23].

## Results

### User Statistics

**Figure 2:**
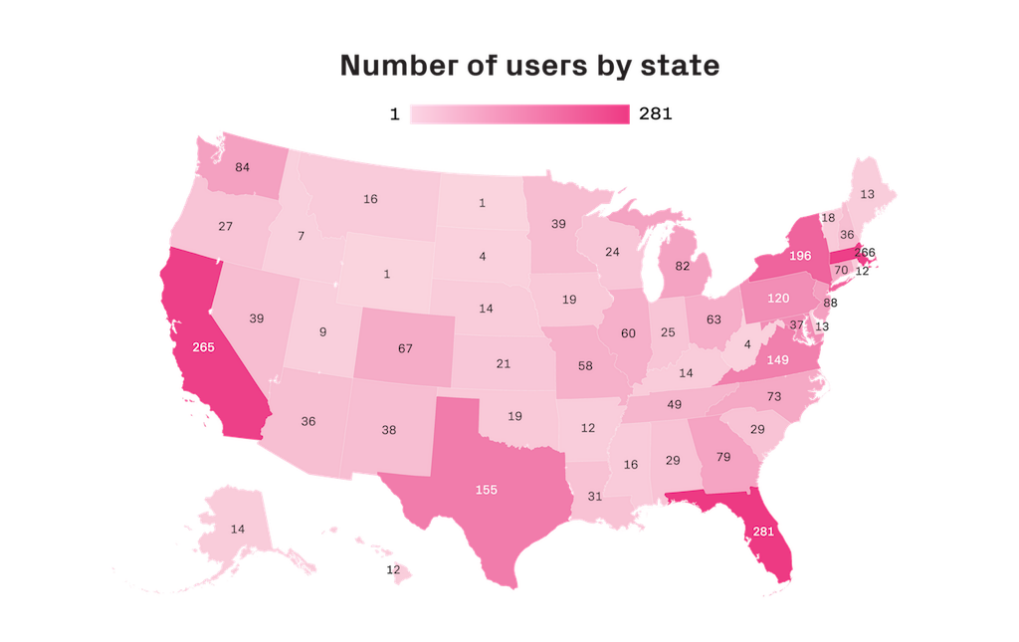
Number of Constant Therapy users by state.

Data for 2850 patients with stroke or TBI endorsing a language or cognitive deficit were included in the analysis. The demographic information for the study sample is presented in Table 1. The majority of patients had a stroke diagnosis (N=2213), had disease onset less than or equal to 6 months before initiating digital therapy (N=1692), and lived in a nonrural area (N=2624). A map depicting the number of total patients by US state is presented in Figure 2. The average age of a patient with stroke was 64.65 (SD 13.15) years, whereas the average age of a patient with TBI was 49.28 (SD 17.80) years, and both diagnoses had a slightly higher proportion of patients who were male (1633/2850, 57.39% and 1664/2850, 58.39%, respectively). The average user completed 18.60 weeks of therapy (range 1-53 weeks) at a frequency of 1.5 days per week (range 0.50-4.77). During their first 20 weeks of access to the Constant Therapy program, patients completed a total of 37 therapeutic sessions on average (range 1-890 sessions).

**Table 1:** Descriptive statistics of users (N=2850).

Characteristic			Values
**Demographic**			
	Age (years), mean (SD)		61.22 (15.69)
**Age group (years), n (%)**			
	≤50	638 (22.39)	
	51-70	1339 (46.98)	
	>70	873 (30.63)	
Female, n (%)			1208 (42.38)
**Condition, n (%)**			
	≤6 months	1692 (59.36)	
Stroke diagnosis, n (%)			2213 (77.65)
Rural location, n (%)			226 (7.93)
**Self-reported deficits, n (%)**			
	Difficulty understanding written language	1959 (68.74)	
	Difficulty understanding spoken language	388 (13.61)	
	Difficulty speaking	2068 (72.56)	
	Difficulty writing	1769 (62.07)	
	Difficulty remembering or retrieving information	2055 (72.11)	
	Difficulty with attention	1688 (59.23)	
	Difficulty processing visual details	1431 (50.21)	
	Difficulty with problem solving	1808 (63.44)	
	Difficulty with executive functioning	445 (15.61)	
**Use of digital therapy, mean (SD)**			
	Number of weeks of use	18.60 (14.68)	
	Average active days per week	1.49 (0.48)	
	Number of sessions	37.00 (47.96)	

### Outcome Evaluation

Results from linear regression models (Table 2) demonstrate that stroke or TBI diagnosis and gender do not have a statistically significant effect on the total number of active weeks of therapy, the average number of active days per week, or the total number of sessions completed during the first 20 weeks of program access. Across all activity metrics, the impact of having a chronic condition (>6 months from disease onset) had a significant effect on the level of therapeutic engagement. After controlling for age, gender, diagnosis, and geographic location, chronic patients completed 4.58 more weeks of therapy (*P*<.001) and 4.53 more sessions (*P*=.02) during their first 20 weeks of access than patients with an acute condition. However, patients with chronic TBI or stroke had a lower frequency of therapy, with −0.10 fewer days per week than acute patients (*P*<.001).

Age exerted different effects across the three activity measures. Specifically, age group was not a significant predictor in determining the total number of weeks of therapy; however, patients older than 70 years had 0.1 fewer average active therapy days per week (*P*<.001), and patients aged between 51 and 70 years completed 5.01 more sessions during their first 20 weeks of program access (*P*=.04) compared with younger patients (aged ≤50 years).

After controlling for all model covariates, patients living in a rural location had a higher frequency of therapy than their urban counterparts, with 0.06 (*P*=.05) more active days per week. Furthermore, rural patients completed 11.54 (*P*=.001) more sessions during the first 20 weeks of access to digital therapy than patients living in an urban setting, after controlling for age, gender, diagnosis, and chronicity. Rural location did not have a statistically significant impact on the total number of weeks of therapy; therefore, a patient’s location was not necessarily a barrier to obtaining the desired duration of therapy.

**Table 2:** Digital therapy usage regression results (N=2850).

Model component^a^	Number of weeks	Active days per week	Number of sessions
Intercept, beta (95% CI)	14.88 (13.26 to 16.5)^b^	1.57 (1.52 to 1.62)^b^	29.53 (24.19 to 34.88)^b^
Male, beta (95% CI)	1.06 (−0.02 to 2.14)	−.01 (−0.04 to 0.03)	.89 (−2.66 to 4.46)
Stroke (vs traumatic brain injury), beta (95% CI)	.47 (−0.92 to 1.85)	.01 (−0.04 to 0.05)	2.33 (−2.25 to 6.91)
Chronic condition, beta (95% CI)	4.58 (3.47 to 5.69)^b^	−.1 (−0.14 to −0.07)^b^	4.53 (0.88 to 8.18)^c^
Rural, beta (95% CI)	1.23 (−0.74 to 3.2)	.06 (0 to 0.13)	11.54 (5.04 to 18.04)^d^
Age 51-70 years^e^, beta (95% CI)	1.37 (−0.06 to 2.81)	−.02 (−0.07 to 0.02)	5.01 (0.29 to 9.73)^c^
Age ≥71 years^e^, beta (95% CI)	.46 (−1.13 to 2.04)	−.11 (−0.16 to −0.06)^b^	.10 (−5.11 to 5.32)
*R* ^2^	0.026	0.018	0.009

^a^Model intercepts are interpreted as the average level of activity for a given individual, independent of their age, gender, location, diagnosis, or time since injury.

^b^*P*<.001.

^c^*P*<.05.

^d^*P*<.01.

^e^Comparison group: age 50 years or less.

### Sensitivity Analysis

**Figure 3:**
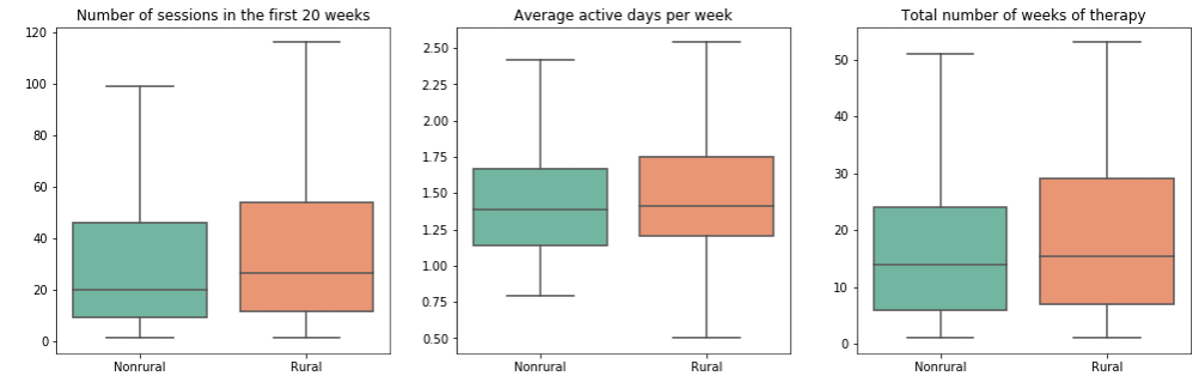
Propensity score–matched rural and nonrural sample (N=452).

ANOVA results from a balanced, propensity score–matched sample of urban and rural patients (N=226 per sample) confirmed that rural patients completed statistically significantly more sessions during their first 20 weeks of access to the Constant Therapy program (47.49 vs 34.46; F_1,521_=4.52; *P*=.03); however, the number of active days per week was not statistically different between the 2 groups (1.55 vs 1.47 days; F_1,521_=2.29; *P*=.13; Figure 3). Similar to the multiple regression analysis on the full sample, the number of active weeks of therapy was not statistically different between rural and urban patients in the propensity score–matched sample (19.73 weeks vs 17.69 weeks; F_1,521_=2.21; *P*=.14).

## Discussion

### Principal Findings

Technological adoption among elders in the United States has been increasing in recent years, with the proportion of adults aged 65 years or older who own a smartphone increasing from 18% in 2013 to 42% in 2016. However, the rate of adoption remains markedly lower than that of the younger population (79% for people aged 50-64 years, 92% for people aged 30-49 years, and 96% for people aged 18-29 years) [18]. Similarly, adoption among Americans living in a rural area has also been consistently lower, with 71% smartphone ownership and 49% tablet ownership, compared with 83% smartphone ownership and 58% tablet ownership among suburban dwellers in 2019 [19]. In contrast to these general trends, we found that among patients using tablets or smartphones for rehabilitation therapy, older patients were just as engaged as younger patients in terms of the duration of therapy and in fact completed more therapeutic sessions during their first 20 weeks of access to the Constant Therapy program. We specifically see that patients aged 51 to 70 years completed more sessions during their first 20 weeks of program access than patients aged 50 years or younger. These findings suggest that older patients who experience neurological injury, which make up the majority of the patients in our sample, are highly likely to engage in digital therapy and are motivated to practice. Recent analyses have concluded that older age is associated with lower effort and self-reported motivation for rehabilitation in both stroke and TBI populations [24,25]. In the case of patients with TBI, the point of declining effort was seen to be as early as 44 years. These findings focused on in-clinic rehabilitation shortly after injury (ie, 1-4 weeks). The fact that both groups over the age of 50 years trended toward higher usage during their first 20 weeks of program access (when compared with those aged ≤50 years) suggests that motivation and support for rehabilitation may present differently in the home-based environment of digital therapy.

Patients who live in a rural location also engaged in more therapeutic sessions and were active more days per week than urban or suburban users, with results on total therapeutic sessions being robust to propensity score–matched sample comparisons. Therefore, although there may be barriers for individuals in rural areas to access technology-based health care solutions initially, those who do are actively engaged and can benefit from digital therapy. Our analysis does not take socioeconomic status into account and, therefore, does not suggest a geographical difference in the ability to afford technological products or subscription-based digital therapy. Given that all patients in our sample were able to access the required technology and therapeutic program, our results are best interpreted in the framework of access to in-clinic services, which may be more difficult for rural users, given proximity and travel requirements. A further analysis of our data demonstrated that the frequency of patients with Constant Therapy accounts set up by clinicians was significantly lower among rural users (*χ*^2^_1_=4.5; *P*=.03) when compared with patients in an urban setting. Therefore, digital rehabilitation may allow rural users to engage in therapy at a frequency that is similar to the patients who have an easier time accessing clinical services.

Patients with a chronic condition (injury >6 months from program initiation) completed more sessions and engaged in therapy for more weeks than acute patients, regardless of age, gender, diagnosis, or geographic location. However, similar to previous analyses that evaluate the amount of therapy received by time since injury, chronicity was also associated with significantly fewer active therapy days per week [26]. These 3 results suggest that although acute patients practice digital therapy more frequently, perhaps because of the functional gains associated with early rehabilitation [27], patients in the chronic phase of recovery participate in a rehabilitation program that is longer in duration and includes more therapeutic sessions on days with active therapy. In addition, platform usage did not differ by neurologic diagnosis. Although the exact therapeutic approach and exercises assigned to a patient may differ by condition, this result suggests that patients with stroke or TBI are able to access the digital therapy they require in a similar manner.

Finally, the average patient in this retrospective analysis completed 37 therapy sessions during a 20-week period, which is much more than the typical patient in the clinical setting where sessions can be as infrequent as once every 2 weeks [26,28]. Although our analysis did not evaluate effectiveness outcomes, the ability for increased data collection with digital rehabilitation has the potential to help answer clinical questions that require more data than are typically available. Given that multiple factors influence both the level of impairment and degree of improvement for a patient, large amounts of data are required to scalably understand the effect of individualized factors on rehabilitation outcomes [29-31]. Digital therapy can make this scale of data collection and evaluation possible by lowering barriers to access and delivering therapy remotely on a platform that collects data continuously. Furthermore, the use of digital rehabilitation for data collection can serve as a low-cost alternative to traditional clinical trial methods, where high dropout rates can lead to inconclusive results at follow-up [2,32].

### Comparison With Prior Work

Understanding how usage of tablet-based or smartphone-based rehabilitation at home might be affected by a patient’s age, gender, geographic location, diagnosis, and chronicity is important to understand how digital therapeutics might scale to serve a larger population. Previous publications that examine the usage patterns of digital rehabilitation for SLT have generally been in a setting where a curated therapeutic schedule was suggested or prescribed. Although this structure is needed to determine effectiveness, these studies do not necessarily provide insight into how digital rehabilitation would be adopted as it becomes more readily available. Specifically, current statistics for digital therapy tend to reflect usage within the context of research studies, which may be more limited by protocols than observational data. For instance, a recent publication by Kurland et al [11] evaluated the effectiveness of a tablet-based treatment program for 21 patients with chronic aphasia over a 6-month period. Compliance to the suggested regimen (5 days a week for at least 20 min) was 83%. However, practice time was self-reported, and it is unclear how the observed usage might differ if a predetermined frequency of practice was not explicitly recommended. Similarly, a recently completed clinical trial (Big CACTUS) evaluated the effectiveness of computerized word finding training in 285 adults with chronic aphasia who used the digital therapy 20 to 30 min a day for 6 months with monthly volunteer support [33]. Although it was noted that 61% of the patients used the software beyond the 6-month protocol, statistics on their usage in an observational context has not been reported [34].

### Limitations

There are some important caveats to this retrospective analysis. First, although the Constant Therapy platform allows for the collection of a large amount of data across several English-speaking countries, it is currently impossible to collect detailed demographic information from all individuals. Specific to the work presented here, educational status and baseline severity were not collected upon account creation. Previous research has shown that both these factors can impact the functional outcomes of neurologic rehabilitation [29]; therefore, their exclusion creates an omitted variable bias, and it is unclear how our results might have changed with their inclusion. In addition, measures of session activity that may have further differentiated our sample, such as the number of items completed during a therapeutic session or the length of time of a therapeutic session, were not explored. However, the three measures used in this analysis are intended to be generalizable across patients, given that the actual content of each therapeutic session will vary based on individual patient needs. Reasons for therapy discontinuation (eg, cost of the program and deficit improvement) and the effect of deficits that present potential barriers to technological usage (eg, difficulty processing visual details) were also not evaluated.

Demographic information collected upon account creation, including age and diagnosis, are self-reported and not verified by a clinician. Furthermore, several pieces of data were not available in our sample, including deficit severity, information on nonvirtual therapy support (eg, in-clinic visits for SLT and support from family members or caregivers), familiarity with technology, and technological failures during use (which may influence a patient’s usage of digital therapy). Our analysis only evaluates the usage of digital therapy in general but does not attempt to define whether exercises were completed accurately or have an impact on clinical outcomes, which would require standardized measures of cognitive and speech improvement to be administered to the sample.

Although important for determining the possible range of digital therapy use, patients with low utilization rates (eg, 1-2 sessions) may not be indicative of the broader population of stroke and TBI patients who have adopted rehabilitation technology for home practice. A sensitivity analysis in which patients were required to have at least ten therapeutic sessions resulted in the same statistically significant results presented in Table 2, with the exception of the third activity metric, where age 51 to 70 years (*P*=.09) and chronic condition (*P*=.06) lost statistical significance for predicting the total number of sessions completed during the first 20 weeks of program access.

Finally, geographic location was approximated by the IP address of the account at sign up, which may differ from the residential address of the user associated with the account. Our sample had a lower representation of rural users than the general US population (7.93% Constant Therapy users vs 19.23% US population) [35], which most likely reflects known disparities in technological adoption rates. The aim of our analysis was to evaluate therapeutic engagement after a patient acquired access to digital therapy; therefore, our study sample represents a population that most likely has a higher likelihood of technological adoption than the general population.

### Conclusions

An evaluation of real-world data demonstrated that patients with stroke and TBI used digital therapy frequently for cognitive and language rehabilitation at home. Digital therapy usage was higher in areas with limited access to clinical services and was not affected by typical barriers to technological adoption, such as age. Moreover, patients in the chronic stage of recovery (who generally face more hurdles in receiving therapy) were engaged in active therapy for longer than those in the acute stage of recovery. These findings will help guide the direction of future research in digital rehabilitation therapy, including the impact of demographics on recovery outcomes and the design and recruitment of large, randomized controlled trials.

## Abbreviations

ANOVA: analysis of variance
IP: Internet Protocol
IRB: Institutional Review Board
SLT: speech and language therapy
TBI: traumatic brain injury
